# Willingness to use and pay for options of care for community-dwelling older people in rural Vietnam

**DOI:** 10.1186/1472-6963-12-36

**Published:** 2012-02-14

**Authors:** Le Van Hoi, Nguyen Thi Kim Tien, Nguyen Van Tien, Dao Van Dung, Nguyen Thi Kim Chuc, Klas Goran Sahlen, Lars Lindholm

**Affiliations:** 1 Umeå Centre for Global Health Research, Department of Public Health and Clinical Medicine, Umeå University, S-901 85 Umeå, Sweden; 2 Hanoi Medical University, 01, Ton That Tung Street, Hanoi City, Vietnam; 3 Central Health Information and Technology Institute, 135 Alley, Nui Truc Street, Hanoi City, Vietnam; 4 Ministry of Health, 138A, Giang Vo Street, Hanoi City, Vietnam; 5 National Assembly Committee for Social Affair, 35, Ngo Quyen Street, Hanoi City, Vietnam; 6 Central Committee of Communication and Education, 07, Nguyen Canh Chan Street, Hanoi City, Vietnam; 7 Ageing and Living Conditions Programme, Centre for Population Studies, Umeå University, S-901 85 Umeå, Sweden; 8 Department of Nursing, Umeå University, S-901 85 Umeå, Sweden

## Abstract

**Background:**

The proportion of people in Vietnam who are 60 years and over has increased rapidly. The emigration of young people and impact of other socioeconomic changes leave more elderly on their own and with less family support. This study assesses the willingness to use and pay for different models of care for community-dwelling elderly in rural Vietnam.

**Methods:**

In 2007, people aged 60 and older and their family representatives, living in 2,240 households, were randomly selected from the FilaBavi Demographic Surveillance Site. They were interviewed using structured questionnaires to assess dependence in activities of daily living (ADLs), willingness to use and to pay for day care centres, mobile care teams, and nursing centres. Respondent socioeconomic characteristics were extracted from the FilaBavi repeated census. Percentages of those willing to use models and the average amount (with 95% confidence intervals) they are willing to pay were estimated. Multivariate analyses were performed to measure the relationship of willingness to use services with ADL index and socioeconomic factors. Four focus group discussions were conducted to explore people's perspectives on the use of services. The first discussion group was with the elderly. The second discussion group was with their household members. Two other discussion groups included community association representatives, one at the communal level and another at the village level.

**Results:**

Use of mobile team care is the most requested service. The fewest respondents intend to use a nursing centre. Households expect to use services for their elderly to a greater extent than do the elderly themselves. Willingness to use services decreases when potential fees increase. The proportion of respondents who require that services be free-of-charge is two to three times higher than the proportion willing to pay full cost. Households are willing to pay more than the elderly for day care and nursing centres. The elderly are more willing to pay for mobile teams than are their households. Age group, sex, literacy, marital status, living arrangement, living area, working status, poverty, household wealth and dependence in ADLs are factors related to willingness to use services.

**Conclusions:**

Community-centric elderly care will be used and partly paid for by individuals if it is provided by the government or associations. Capacity building for health professional networks and informal caregivers is essential for developing formal care models. Additional support is needed for the most vulnerable elderly to access services.

## Background

The world's elderly population has been growing for centuries. With the increase in life expectancy that followed improvement of health care and nutrition over the last century, this portion of the population is increasing more rapidly. The growth rate of older people in developing countries began to rise in the early 1960s and has doubled that of developed countries and the total world population [1]. In 2007, the number of people aged 60 and older was 793 million worldwide, and accounted for 11.7% of the world population [2]. This number is projected to increase to almost two billion by 2050 [1].

Vietnam has experienced rapid aging with declines in both fertility and mortality [3]. The proportion of people aged 60 years and older within the general population increased from 6.7% in 1979 to 9.2% in 2006 [4], and is projected to be 26.1% by 2050 [5]. The Vietnamese ageing index, the number of persons 60 years or older per hundred persons under age 15, increased from 18.2% in 1989 to 37.0% in 2007 [6]. At the same time, the old-age dependency ratio (the number of persons 60 years and over per one hundred persons 15 to 59 years) increased from 0.13 in 1989 to 0.15 in 2007 [7]. The total population of Vietnam reached 85 million in 2007, with 72.6% residing in rural areas [8]. The proportion of older people within the rural population increased from 7.4% in 1989 [9] to 10.3% in 2006 [10]. The rural elderly population accounted for 73.3% of the total elderly population in 2004 [11].

Among older people, there are more females than males. This sex imbalance is higher in rural than urban areas. The proportion of people aged 60-74 years among older people decreased while that of people aged 75 and over increased [11]. Most of older people are married or widowed. Divorce and permanent separation are relatively uncommon. Just over one-fourth of the elderly profess a religion. Three-fifths of them are Buddhist and approximately 20% are Catholic. The rural elderly are disadvantaged in terms of educational attainment, housing quality, access to media [11], poverty status [12], and access to health care [13].

A majority of the elderly are household heads and live with a child. Only 11.5% live with a spouse alone, and 5.8% live alone. Living with a child is more common in urban areas while living alone or with only a spouse is more common in rural areas. Most of those who live alone are women or living in rural areas [11]. The elderly, especially in rural areas, are more likely to rely on domestic sources of economic support than on the social security system [14] because the rural elderly are less likely to have paid into the social insurance system. There is an increasing trend of temporary migration from rural to urban areas among the young because of better employment opportunities [15]. This leaves more elderly living on their own with less physical and emotional support from family members [16].

Half of the rural elderly and one-third of the urban elderly remain economically active for a paid salary, in the household's agriculture production, or in other enterprises [11]. Older people's incomes are mainly from agriculture, earnings, trade or other business. Remittances (16%) and formal state transfers (11%) often account for part of the income. Rural people are less likely to live in households that receive formal transfers than are urban people. People living in households receiving social insurance pensions or social subsidies are twice as common in urban areas as rural areas [17] because the rural elderly are more likely to receive welfare payments.

Vietnamese life expectancy at birth increased from 66 to 72 years between1990 and 2006 [18,19], with a projected further increase to 80.3 years by 2050 [5]. There is evidence [3] that life expectancy at age 60 increased overall in rural areas, but decreased among the most vulnerable groups such as older women living without children or grandchildren. Poverty status and living arrangements lead to a wide gap in life expectancy. The gender gap in the life expectancy is consistent across all socioeconomic groups and wider among the more disadvantaged populations.

Health related quality of life (HRQoL) at old age increases in rural areas and varies substantially according to socioeconomic factors [20]. Ageing has a primary influence on HRQoL, which is mainly due to reduction in physical functions rather than mental functions. Being a household head and working during old age are advantageous for attaining better HRQoL in physical but not psychological terms. Economic conditions affect HRQoL through sensory rather than physical roles. Long-term living conditions are more likely to affect HRQoL than are short-term economic conditions.

Although greater life expectancy in old age is an indicator of successful ageing [21], it also means that more elderly suffer from non-communicable diseases (NCDs) [22]. Vietnam is a country where the prevalence of NCD increases at older ages [23,24]. The most common NCDs among elderly Vietnamese are cardiovascular diseases, diabetes, kidney disease, and cancer [25]. The elderly also suffer from accidents, frequent illnesses and multiple or concurrent health disorders. A survey found that 60% of older people were ill during the prior four weeks and 70% suffered from NCDs [25]. Using the INDEPTH WHO-SAGE questionnaire, elderly health among Vietnamese people is higher than among Bangladeshis, but lower than among Indonesians [26].

Health sector reform in Vietnam was initiated in 1989 with the introduction of user fees for public services and development of a private sector. Consequently, household health expenditures now consist predominantly of out-of-pocket payments that accounted for 67% of health expenditures in 2005 [27]. Disparities in health and health care are widening between socioeconomic groups, and between rural and urban areas, despite efforts by the government to improve access to health care. In particular, the rural elderly have less access to care than those in urban areas [13]. Elderly access to health services is often limited by mobility and an inability to afford healthcare services, especially for those who require prolonged care [25].

Among rural elderly, dependence in instrumental or intellectual activities of ADLs is more common than in basic ADLs [28]. Almost one-third of rural older people need help in one or more specific ADLs. At least one-third of those who need help do not receive enough support. Children and grandchildren are the main caregivers of elderly ADLs. Age group, sex, educational level, marital status, household membership, working status, household size, living arrangement, residential area, household wealth, poverty status, and chronic illnesses are determinants of the daily care needs in old age.

The ordinance on elderly people issued in 2003. It was replaced by the law on elderly people in 2010. The law indicates that health care should be developed for elderly people in the community. Commune health centres and local authorities are key stakeholders responsible for organizing the care. Health insurance for all was determined as a main mechanism of health financing. The government is in the process of expanding its support for accessing health insurance by the poor and other vulnerable groups. Currently, people aged 95 years and over are covered by the support.

Responding to the unmet daily care needs of older people, community-centric long-term care was initiated recently to meet the individualized needs of the elderly. However, there is still limited knowledge about the demands of the elderly and their families for various models of care. This is especially true in rural settings. This study was conducted to assess willingness to use (WTU) and willingness to pay (WTP) for particular models of care, and their socioeconomic determinants among elderly and their families in a rural setting. Such data can provide evidence for the design of appropriate health and social policies in Vietnam.

## Methods

### Study setting and the FilaBavi surveillance system

The study was conducted in 2007 within the longitudinal demographic and health surveillance system of FilaBavi [29]. The field site operates in the rural Bavi district of Vietnam. Bavi covers an area of 410 km^2^, including lowland, highland and mountainous areas. Thirty percent of the land is used for agriculture and 17% is forest. Mountainous areas account for 42% of the land mass. The 2007 population was 262,763 people. Among adults over 20 years of age, the majority completed primary/secondary school (65% of men, 73% of women) and high school/higher education (34% of men, 23% of women). The rest are illiterate. Two-thirds of the population are farmers (39% of men, 57% of women) or industrial workers (31% of men, 9% of women) and the remainder are business people, students, government staff, retired persons or others.

The FilaBavi surveillance system consists of a representative sample of 67 out of 352 clusters in the district that have been selected with a probability proportional to size since 1999. In 2007, 53,927 individuals were followed by FilaBavi, and this represented 20.5% of the total district population. People aged 60 and over represented 11.5% of the total population followed by FilaBavi at the 2007 mid-year point.

### Study design, sampling and sample size

The sample size was calculated by estimating a proportion in a population-based survey. Using an estimated proportion of 13% (estimation error of 2.6%) of elderly who need daily support for care in daily living in a rural area of Vietnam [30], a sample size of 2,699 elderly is required. This was adjusted for a design effect of two for cluster sampling of FilaBavi, then doubled for robustness of multivariate analysis, and allows for a 10% non-response rate. This figure is approximately equal to 50% of all people aged 60 or greater in the FilaBavi sampling frame.

Fifty per cent of households with elderly members followed by FilaBavi were randomly selected for a household cross-sectional survey. This resulted in 2,255 households with 2,968 individuals. During the survey period from July to October 2007, 166 households were excluded due to absence of the elderly. However, each of these households was replaced with the nearest unselected household that had elderly members. In total, 2,240 households with 2,873 elderly were included in the quantitative part of this study.

Qualitative data were collected to increase credibility and provide further understanding of the quantitative study. Four focus group discussions (FGD) were conducted in one commune with a socioeconomic status at the district average. The first discussion was with six elderly people, and the second discussion was with six representatives of households with older people. The discussions were organized at one village in the commune. The discussants in each group had equal numbers of men and women.The elderly who participated in the FGD belonged to different age groups. The household representatives were not elderly themselves and had different household roles (two heads, two main caregivers, and two other members).

Six or seven representatives of the key social stakeholders in elderly care participated in each of the other discussions, one at the village level and another at the communal level. These participants included individuals from the local authority, health sector, elderly association, women's union, youth union and former soldiers' union.

### Study variables and information

Willingness to use and willingness to pay for different community-centric models of elderly care were examined among both older people and their household representatives in the cross-sectional survey. Willingness to use each of the models was further considered as a dependent variable in the analysis of its association with a number of independent variables, such as the elderly's need of help in ADLs and socioeconomic characteristics of older people and their households.

Individual characteristics of older people were collected from the survey. These included date of birth, sex, education, marital status, household head status, status of living with spouse, and working status such as working in one's own rice fields or not working. Variables on household economic and living conditions were extracted from the mid-2007 FilaBavi census in order to estimate the wealth index and poverty status of households. This type of census has been repeated every second year since the establishment of FilaBavi in 1999. Variables include land area, structural housing components, assets, sanitation conditions, income, expenditures and debt.

The qualitative study focused on perceived care needs of the elderly, current and expected roles of different key stakeholders, encouraging/limiting factors in providing needed care, solutions for overcoming barriers in providing the care, and expected future models of care.

### Variables measurement and data collection

Using structured questionnaires, face-to-face interviews were performed by 52 trained field FilaBavi personnel at houses with elderly members. Questions included those about supports needed in ADLs, models of elderly care, individual and household characteristics of the elderly.

Three scales of ADLs were applied when measuring the daily care needs. They included Katz's basic ADLs [31] (bathing, dressing, toilet use, transferring in and out of bed or chair, urine and bowel continence, eating), instrumental ADLs (cleaning house, cooking, shopping, travelling) and intellectual ADLs (writing, reading, listening to radio, watching TV). Support needs for each activity (none, need some helps, complete dependence) were assessed, together with levels of support received (none, not enough, enough).

Three options for possible care models were described to older people and the household representatives. These included: a) a mobile team of nurses in the respondent's commune to provide home care services for the elderly at their request; b) a day care centre in the village that would be a place the elderly could visit for a period of time every day or every other day; c) a nursing centre in the commune or district where the elderly could stay for as long as needed (days, weeks or months).

The assumptions for the last two models were that food would be served, relaxation activities provided, and nursing available. For each model, the elderly and their household representatives were asked whether they would likely use the model if it was provided free-of-charge, for a fee (less than the actual cost) or actual cost. In the first two models, expected types of services were listed as choices. Willingness to pay for, and frequency of using services, were asked for each model.

Six field supervisors reviewed each completed questionnaire and randomly selected 5% for re-interview. Each questionnaire with missing or irrelevant values was returned to the field personnel for checking and completion after re-visits to the corresponding households. Double data-entry was performed using EpiData 3.1 http://www.epidata.dk to check for consistent values of each variable. Correction of data-entry errors was based on actual values from the completed questionnaires.

Assets were classified by category, eg, furniture, communication and electrical equipment, types of vehicles, agricultural machines, and cattle. These items were classified as "present" or "not present" regardless of their quantity and quality. Sanitation conditions were assessed as sources of water for drinking and cooking, type of latrine, and presence of a bathroom. All types of income (ie, agriculture, breeding, forestry, and others) were recorded and summed for the total income of a given household. The sum of daily food expenditures was multiplied by 30 days and added to the sum of other monthly expenditures to estimate total monthly household expenditures. Monthly income and expenditures were then divided by household size to generate "per capita" variables.

Using corresponding guidelines, the discussions and interviews were conducted by a main researcher and 1-2 assistant researchers who were trained and experienced with qualitative research methods. The discussions and interviews were manually noted, tape-recorded, transcribed, and translated to English.

### Statistical analysis

The datasets from the present survey and the repeated census were linked and analysed using STATA 10 (StataCorp LP, College Station, TX, USA). An index was calculated for each ADL scale by summing up the score from each activity (score is 0 if no need or need some help; score is 1 if complete dependence). The basic ADL index ranges from 0 to 6. The instrumental and intellectual indices range from 0 to 4. Household wealth index was calculated as the first component for all economic variables from the census. Classification of household wealth quintile was based on the hierarchies among all FilaBavi households. Household poverty status was classified using the national poverty line for rural areas, and based on monthly per capita income being equal to VND 200,000 (USD 12.5) for 2006-2010 [32].

Distributions of study subjects by socioeconomic group, willingness to use care models, frequency of using services, and type of expected service were described using percentages and corresponding 95% confidence intervals. Willingness to pay for care services was estimated as the average monthly expenditure in VND for the elderly or their households with corresponding 95% confidence intervals. Significant differences in percentages or averages between groups of older people, or between older people and their household representatives, were identified by comparing the corresponding 95% confidence intervals.

Multivariate logistic regression analyses were performed to measure the effect of ADL indices and socioeconomic factors on elderly willingness to use care services by models of care and levels of payment. Being independent in ADL, female, aged 80 years and above, illiteracy, widowed status, living without a spouse, position as household member, not working until old age, belonging to the poorest quintile, and living above the national poverty line were used as references in the analyses. A backward stepwise procedure, with a *p*-value of 5% for removal, was used to identify significant factors that remained in the final multivariate model. Robust standard errors from cluster data were used for accurate estimation of the model parameters [33].

### Qualitative analysis

Thematic content analysis was performed by two researchers. Only information that illustrates or explains the quantitative research results regarding care for the elderly is used in this article.

### Ethical considerations

Ethical approval for the FilaBavi demographic surveillance system, including data on socioeconomic status, was given by the Research Ethics Committee at Umeå University, Sweden (reference number 02-420). The present study was also approved by the Research Ethics Committee at Hanoi Medical University (reference number 51/HMU-RB). As all selected households belonged to the sampling frame of FilaBavi DDS, and these individuals were familiar with the DSS data collection, only oral consent was required. Purposes of the study and the main contents of the interviews were briefly described, together with a commitment to keeping individual and household information confidential. The participants reserved the right to refuse to answer any question or withdraw from the interview at any time.

## Results

Table 1 summarizes socioeconomic characteristics of the elderly and their general health status that are described in detail elsewhere [3,20]. The elderly are predominantly women, younger old, literate, married or widowed, living without a spouse, living with children or grandchildren, belonging to households in the middle to richest wealth quintiles, and living above the national poverty line. It is notable that some married couples live in different children's houses, ie, they live separately. Distributions of older people by ADL indices and need of support are documented elsewhere [28].

**Table 1:** Distribution of the elderly by socioeconomic group

*Variables*	*n*	*%*	*95%CI*
*Age groups (years)*			
60-69	1,208	42.0	40.2-43.9
70-79	1,076	37.4	35.7-39.2
80-89	513	17.9	16.5-19.3
90+	76	2.7	2.1-3.2

*Sex*			
Men	1,056	36.8	35.0-38.5
Women	1,816	63.2	61.5-65.0

*Education*			
High school and above	220	7.7	6.7-8.6
Primary/secondary school	1,122	39.1	35.5-37.0
Read and write only	1,012	35.2	37.3-40.9
Illiterate	518	18.0	16.6-19.4

*Marital status*			
Married	1,569	54.8	33.0-56.6
Widowed	1,225	42.8	41.0-44.6
Separated, divorced, or single	70	2.4	1.9-3.0

*Living with spouse*			
Yes	876	30.5	28.8-32.2
No	1,994	69.5	67.8-71.1

*Living with grandchildren*			
Yes	1,689	58.9	57.1-60.7
No	1,181	41.2	39.4-43.0

*Living alone*			
Yes	273	9.5	8.4-10.6
No	2,597	90.5	89.4-91.6

*Household head*			
Yes	1,493	52.1	50.2-53.9
No	1,374	47.9	46.1-49.7

*Working status*			
Yes	1,160	40.4	38.6-42.2
No	1,713	59.6	57.8-61.4

*Living area*			
Lowlands	866	30.1	28.5-31.8
Highlands	1,430	49.8	49.1-51.6
Mountainous	577	20.1	18.6-21.6

*Wealth quintile*			
Richest	603	21.0	19.5-22.5
Richer	639	22.2	20.7-23.8
Middle	637	22.2	20.7-23.7
Poorer	496	17.3	15.9-18.7
Poorest	498	17.3	16.0-18.7

*National poverty line*			
Above	2,445	85.1	83.3-86.4
Below	428	14.9	13.6-16.2

The elderly and their household willingness to use care services by model are presented in Table 2. Use of care from a mobile team was chosen most often. Fewer respondents intend to use a nursing centre. "We will take part in these activities enthusiastically", said one household member in a focus group. Household heads expect to use services to a greater extent for their elderly than the elderly themselves did for all models and payment levels. In a focus group, a 70 year old man stressed that children must take responsibility for their parents and he was doubtful whether society would organize a nursing centre.

**Table 2:** Willingness to use care services among the elderly and their household representatives

Levels of payment/Models of care	*Free-of-charge*		*Less than cost*		*Full cost*	
	%	95%CI	%	95%CI	%	95%CI
*Mobile team*						
Elderly	83.2	81.8-84.6	56.3	54.4-58.1	37.0	35.2-38.8
Household	88.7	87.2-90.1	69.0	66.9-71.1	47.0	44.7-49.2
*Day care centre*						
Elderly	69.7	67.0-71.4	52.0	50.1-53.8	34.7	32.9-36.4
Household	86.0	84.4-87.5	70.9	68.9-73.0	49.4	47.1-51.7
*Nursing centre*						
Elderly	40.7	38.8-42.5	23.6	22.0-25.2	14.9	13.6-16.3
Household	48.6	46.3-50.9	32.8	30.6-34.9	21.7	19.8-23.6

Willingness to use the services decreased when potential fees were higher. As a 68 year old daughter said, "If we have to contribute money, we will do that. However, we can only pay a little." Almost two-fifths of the elderly were willing to use mobile team care at full cost. But only one third were willing to pay full cost for day care centre services, and only 15% for nursing centre services. The proportion of respondents who expect to use care if it is free-of-charge is much higher than those willing to pay at full cost: 1.7-2.2 times higher for household representatives, and 2.0-2.7 times higher among the elderly.

Willingness to use services by ADL index is presented in Table 3. People who are dependent in instrumental ADLs are more likely to use free-of-charge services from a mobile team. Those dependent in basic ADLs are less likely to use services from a day care centre at any levels of payment. Those dependent in intellectual ADLs are less likely to use services from any care model at any level of payment. The exception was free-of-charge services from a mobile team. People are less likely to use services at higher levels of payment from all the models of care.

**Table 3:** Willingness to use care services among the elderly by level of payment, model of care and ADL index

Level of payment/Model of care/ADL index	*Free-of-charge*		*Less than cost*		*Full cost*	
	%	95%CI	%	95%CI	%	95%CI
*Mobile team*						
Basic index = 0	83.2	81.8-84.6	56.3	54.5-58.2	37.1	35.3-38.9
Basic index ≥ 1	82.6	71.2-94.0	52.2	37.2-67.2	34.8	20.5-49.1
Instrumental index = 0	78.7	76.0-81.3	54.0	50.8-57.2	35.1	32.0-38.2
Instrumental index ≥ 1	85.4	83.8-87.1	57.4	55.1-59.7	38.0	35.8-40.2
Intellectual index = 0	82.2	80.1-84.4	63.3	60.6-66.1	40.9	38.2-43.7
Intellectual index ≥ 1	83.9	82.1-85.8	50.7	48.3-53.2	34.0	31.6-36.3

*Day care centre*						
Basic index = 0	70.5	68.8-72.2	52.6	50.8-54.5	35.1	33.3-36.9
Basic index ≥ 1	19.6	7.7-31.5	13.0	2.9-23.2	8.7	0.2-17.2
Instrumental index = 0	71.9	69.0-74.8	55.0	51.8-58.2	36.5	33.4-39.6
Instrumental index ≥ 1	68.6	66.5-70.7	50.5	48.2-52.7	33.7	31.6-35.9
Intellectual index = 0	80.7	78.5-82.9	66.8	64.2-69.5	44.7	41.9-47.5
Intellectual index ≥ 1	61.1	58.7-63.5	40.4	38.0-42.8	26.8	24.6-29.0

*Nursing centre*						
Basic index = 0	40.9	39.0-42.7	23.8	22.2-25.4	15.1	13.7-16.4
Basic index ≥ 1	28.3	14.7-41.8	13.0	2.9-23.2	8.7	0.2-17.2
Instrumental index = 0	39.4	36.2-42.6	21.8	19.1-24.5	13.1	10.9-15.3
Instrumental index ≥ 1	41.3	39.1-43.6	24.5	22.5-26.5	15.9	14.2-17.5
Intellectual index = 0	45.8	43.0-48.6	31.8	29.1-34.4	21.2	18.9-23.5
Intellectual index ≥ 1	36.7	34.3-39.1	17.2	15.4-19.1	10.1	8.6-11.6

The expected frequencies of using services are summarized in Table 4. Most elderly expect to use a mobile team 1-3 times per month. The next largest group expects use to be 1-2 times per day. The second largest group is more dependent and in need of daily services. The most frequently expected use of a nursing centre was 1 week per month, followed by 2-3 weeks per month, and then ≥ 1 month per year. There was no significant difference in expected frequency of use by payment level for use of services in any of the models.

**Table 4:** Expected frequency of using care services among the elderly and their household representatives

Levels of payment/Model of care	*Free-of-charge*		*Less than cost*		*Full cost*	
	%	95%CI	%	95%CI	%	95%CI
***Mobile team***						
*Elderly*						
1-2 times/day	25.6	23.9-27.5	23.7	21.5-25.8	27.1	24.4-29.9
1-6 times/week	19.8	18.1-21.4	18.0	16.1-20.0	15.0	12.8-17.2
1-3 times/month	42.0	40.0-44.1	43.9	41.5-46.4	43.8	40.7-46.8
Other	12.6	11.2-13.9	14.4	12.6-16.1	14.1	12.0-16.2
*Household*						
1-2 times/day	26.6	24.5-28.8	26.4	24.0-28.9	28.9	25.9-31.9
1-6 times/week	17.6	15.7-19.4	14.8	12.8-16.8	12.6	10.4-14.8
1-3 times/month	42.3	39.9-44.7	44.4	41.6-47.1	42.9	39.6-46.2
Other	13.5	11.8-15.1	11.4	12.5-16.3	15.6	13.2-18.0

***Nursing centre***						
*Elderly*						
1 week/month			44.4	40.3-48.5	41.8	36.7-46.9
2-3 weeks/month			23.4	19.9-26.8	26.0	26.0-30.5
1 month/year			17.9	14.8-21.1	16.9	13.1-20.8
> 1 month/year			14.3	11.4-17.2	15.3	11.6-19.0
*Household*						
1 week/month			39.9	35.6-44.1	43.7	38.4-49.1
2-3 weeks/month			22.2	18.6-25.8	20.9	16.6-25.4
1 month/year			18.4	15.0-21.7	18.3	14.1-22.4
> 1 month/year			19.5	16.1-23.0	17.1	13.0-21.1

Opinions on the types of service provided by a mobile team and a day care centre are described in Table 5. The highest demand is for medical examinations from a mobile team; this is followed by health consultation and taking drugs or injections. The least frequently requested services are rehabilitation, assistance with personal hygiene, and eating and drinking. Both household heads and seniors most frequently suggest regular examinations would be used. This need is also supported by association representatives.

**Table 5:** Opinions of the elderly and their household representatives on care services that should be provided

Care services	Elderly		Household	
	%	95%CI	%	95%CI
*Mobile team*				
Medical check up	93.8	93.0-94.8	95.1	94.1-96.1
Health consultation	73.6	72.0-75.3	75.3	73.3-77.3
Taking drugs or injections	53.3	51.5-55.2	53.5	51.2-55.8
Rehabilitation	36.1	34.3-37.8	38.4	36.2-40.6
Personal hygiene	23.5	21.9-25.1	23.1	21.2-25.0
Eating and drinking	23.2	21.6-24.8	23.9	22.0-25.9
*Day care centre*				
Physical exercises	77.4	75.9-79.0	79.1	77.2-80.9
Health consultation	71.1	69.4-72.8	72.7	70.7-74.8
Relaxation	62.6	60.9-64.5	66.3	64.2-68.5
Nursing care	55.0	53.2-56.8	59.9	57.6-62.1
Social interactions	49.6	47.7-51.4	52.5	50.2-54.7
Food and drink	24.6	23.0-26.2	27.0	25.0-29.1

The most demanded day care centre services are physical exercises, health consultations, relaxation and nursing care. The least required services are relationship exchanges and drinks. The elderly and their household representatives have almost the same expectation of service types that would be provided in either care model. One exception was that a higher percentage of household members expect their elders to need nursing care at day care centres than did the elderly themselves.

Willingness to pay for services in each care model is presented in Table 6. Both elders and their households are willing to pay the highest monthly amount for using a nursing centre care but the elderly are only willing to pay the lowest amount for day care centre care use. Their households are willing to pay the same amount for using mobile team or day care centre services. Households are willing to pay higher costs for day care and nursing centre care for their elderly relatives. In contrast, the elderly are willing to pay more for mobile team services than their households are.

**Table 6:** Willingness to pay for care services provided by various care models among the elderly and their household representatives*

Care services	*Elderly*		Household	
	Mean	95%CI	Mean	95%CI
Mobile team	34,192	31,661-36,722	28,296	26,550-30,042
Day care centre	21,148	19,585-22,711	27,929	23,065-32,794
Nursing centre	48,603	43,859-53,346	68,778	59,786-77,771

* VND/month

The multivariate effect of ADL indices and socioeconomic factors on WTU is summarized in Table 7. Older people who are more dependent in intellectual ADLs are less likely to use services from any model of care except free services from a mobile team. Those who are more dependent in instrumental ADLs are more likely to use services from a mobile team or a nursing centre, except if the mobile team is at full cost or the nursing centre is a free service. Those who are more dependent in basic ADLs are less likely to use services from a day care centre at any level of payment.

**Table 7:** Effect of socioeconomic factors on willingness of the elderly to use care services

Levels of payment/Terms	*Free-of-charge*			*Less than cost*			*Full cost*		
	**Coef**.	OR	P	**Coef**.	OR	P	**Coef**.	OR	P
*Mobile team*									
Instrumental ADL index	0.151	1.16	0.001	0.085	1.09	0.018			
Intellectual ADL index				-0.219	0.80	< 0.001	-0.103	0.90	0.003
Aged 60-69				0.188	1.21	0.040			
Married	0.473	1.61	0.001	0.244	1.28	0.026			
Separated/Divorced				-0.648	0.52	0.016			
Living with spouse	-0.797	0.45	< 0.001	-0.582	0.56	< 0.001			
Living with son/daughter				-0.255	0.78	0.029			
Living with grandchildren				0.298	1.35	0.004	0.356	1.43	< 0.001
Lowlands	-0.267	0.77	0.018				-0.328	0.72	< 0.001
Living under NPL				-0.268	0.77	0.018	-0.267	0.76	0.023
Richest quintile	-0.793	0.45	< 0.001						
Richer quintile	-0.451	0.64	0.003	0.249	1.28	0.011			
Middle quintile	-0.368	0.69	0.014				-0.290	0.75	0.004
Poorer quintile							-0.298	0.74	0.007
Constant	1.91	-	< 0.001	0.385	-	0.002	-0.373	-	< 0.001

*Day care centre*									
Basic ADL index	-0.542	0.58	< 0.001	-0.452	0.64	0.001	-0.540	0.58	0.001
Intellectual ADL index	-0.392	0.68	< 0.001	-0.363	0.70	< 0.001	-0.276	0.76	< 0.001
Aged 60-69	0.352	1.42	0.008	0.720	2.06	< 0.001	0.738	2.09	< 0.001
Aged 70-79	0.305	1.36	0.012	0.557	1.75	< 0.001	0.460	1.59	< 0.001
High school & higher				0.488	1.63	0.007			
Primary/secondary school				0.245	1.28	0.014			
Married	0.500	1.65	< 0.001						
Living with spouse	-0.828	0.44	< 0.001	-0.430	0.65	< 0.001			
Living alone				-0.437	0.65	0.002			
Living with grandchildren							0.275	1.32	0.001
Highlands	0.254	1.29	0.004						
Still working				-0.194	0.824	0.030	-0.379	0.69	< 0.001
Living under NPL							-0.325	0.72	0.010
Richest quintile				0.204	1.23	0.001			
Middle quintile	0.277	1.32	0.011				-0.328	0.72	0.002
Poorer							-0.285	0.75	0.015
Constant	0.928	-	< 0.001	0.085	-	0.537	-0.663	-	< 0.001

*Nursing centre*									
Instrumental ADL index				0.159	1.17	< 0.001	0.215	1.24	< 0.001
Intellectual ADL index	-0.179	0.84	< 0.001	-0.367	0.69	< 0.001	-0.438	0.65	< 0.001
Aged 60-69				0.380	1.46	0.018			
Aged 70-79				0.294	1.34	0.046			
Male	0.241	1.27	0.009	0.215	1.24	0.048			
High school & higher	-0.332	0.72	0.044						
Living with spouse	-0.372	0.69	0.001	-0.356	0.69	< 0.001	-0.368	0.69	0.003
Lowlands	-0.482	0.62	< 0.001	-0.636	0.53	< 0.001	-0.847	0.43	< 0.001
Living under NPL							-0.355	0.70	0.040
Richest quintile	-0.624	0.54	< 0.001						
Richer quintile	-0.362	0.70	0.001						
Poorer quintile	-0.457	0.63	< 0.001						
Constant	0.304	-	0.002	-1.085	-	< 0.001	-1.229	-	< 0.001

*Coef *coefficient; *OR *odds ratio; *P p *value; *NPL *national poverty level

Those who are younger elderly, men, married, with educational levels of elementary/secondary school, living with grandchildren, or living in the highlands, state that they are more likely to use care services. Those who are separated/divorced, live with a spouse or alone, live with a son or daughter, live in the lowlands, work until an older age, live under the national poverty line, or are in the poorest quintile are less likely to use services. People with an educational level of high school or higher are more likely to use free services from a day care centre but less likely to use services from a nursing centre.

The elderly in the richest two quintiles are less likely to use free services from a nursing center. Those in the richest quintile are less likely to use free service from a mobile team but more likely to use services from a day care centre requiring partial payment. Those in the wealthiest quintile are less likely to use free services but more likely to use mobile team services requiring partial payment. Respondents who were in the middle wealth quintile were more likely to use free services but less likely to use day care services at full price.

## Discussion

During the Vietnamese transition to a modern society, the need for community-based long-term elderly care is acknowledged by the elderly, their households and representatives from the village and commune levels. This unanimity is the most interesting finding of this study. Willingness to use care services is affected by elderly ADL dependence, socioeconomic status, living arrangement and required payment level. The study respondents were willing to pay for care services at certain levels but indicated that society or associations have a responsibility to provide elderly care. There currently are no comparative figures on the need for community-centric long-term elderly care in Vietnam.

Older people in Vietnam prefer to receive care at home. This is in accordance with tradition and consistent with patterns in other countries [34]. The majority of households and elderly only expect to stay intermittently in a nursing centre, such as 1-3 weeks/month, rather than using more long-term care as do elderly in many developed countries. The frequency of using services from any model was not dependent on the payment levels. This may be because willingness to pay was asked as a monthly payment rather than by episode of service use.

In spite of household representative expectations that their elderly would use services more often than the elderly themselves, the consensus is surprising. The findings indicate a trend of expansion of elderly care from family caregivers to a social network. This trend is likely the result of demographic pressures and socioeconomic transitions within the country. Willingness to use free services was 2-3 times higher than willingness to pay full price. This suggests a large gap between household needs and affordability of care for the rural elderly. Health insurance and medical fee exemption in Vietnam cover 33.5% of older people aged 60-89 and 37.1% of those aged 90 years and above [25]. With this coverage, additional social and health policies to promote the formulation and use of community-centric care models are necessary. Further expanding the support for accessing free-of-charge health insurance for elderly people at younger ages, and including long-term care services in reimbursement schemes of health insurance would compensate for the limited affordability of care.

The elderly expect to receive professional care, including curative, preventive and rehabilitative services from a mobile team more than they expect informal care with tasks of daily living at home. "I suggest to the authorities that there be regular check-ups for old people" was a common view from the elderly. This opinion was supported by village leaders: "The best thing we can do is organize regular check-ups for the elderly". Medical doctors, medical doctor assistants, and nurses, working together with informal caregivers, will be essential for future provision of home care. Current estimates are 5.9 physicians and 5.6 nurses per 10,000 inhabitants in the public and private health sectors [35]. Therefore, capacity building of a network between health professionals and informal caregivers should be addressed in strategies to expand community-centric elderly care. Social associations are also willing to contribute: "If they need builders, our members can help. Moreover, we can contribute some money".

The 2008 Vietnam Household Living Standard Survey [36] estimated that household per capita health care expenditure in rural areas accounts for 7% of the total household per capita expenditure. In the current study, elderly are willing to pay 2-4% of the monthly per capita household expenditure and their families were willing to pay 3-6% for services from each care model. Therefore, the maximum amount that respondents were willing to pay was almost equal to the per capita expenditure for health care.

Higher dependency in instrumental ADLs is related to higher WTU for a mobile team (except at full cost) or nursing centre (unless it is a free service). Dependent people may think they cannot afford the service at full cost price, and free-of-charge services may encourage the expectation of using services from both groups, regardless of need. This suggests that additional supports are needed for rural people to access enough care through future interventions.

A higher intellectual ADL index is associated with a lower WTU from all models of care, except free services from a mobile team. This may be because support for intellectual ADLs are mainly provided by family caregivers [28], rather than using day care, a nursing centre, or paying for services from a mobile team. People who are more dependent in basic ADLs are less likely to use services from a day care centre at any level of payment. This may be explained by the fact that physical exercises, health consultations and relaxation were examples of the services that could be provided in a day care centre rather than nursing care. Or, people in need of personal care preferred or relied on family support, particularly from their sons, daughters or grandchildren rather than from outsiders.

Being younger elderly is associated with interest in using a day care centre regardless of levels of payment. This could be because younger elderly are healthier and eager to participate in health promotion activities. The tendency among men to use more services can be attributed to rural women being more active in the care of grandchildren and housework, while men expect to care for themselves under the patrilineal and patrilocal culture [37] that remains strong in rural Vietnam. The observed trend of using more free services from a nursing centre or services with lower costs from a day care centre among people with low levels of education may be influenced by the lower incomes that are typical of this group [38].

It is notable that only 55.8% of married elderly are living with their spouse. The rest may live with the families of their children. People who live with a spouse are less likely to use services; widowed, divorced or single elders are more likely to use services. This may be explained by the spouse being one of the most important sources of emotional and practical support [39]. Married people have a tendency of using more services that are free-of-charge or with lower costs. This may be affected by the 44.5% of married people who do not live with a spouse. People who live in the lowlands usually have more geographic and economic access to health services than those living in mountainous areas. This may lead to a lower expected use of mobile teams and nursing centres.

The need to spend time generating income as well as a better general health status among people still working at older ages [20] may explain their lower expectation of using day care centre services. People who live under the national poverty line or belong to the lower wealth quintiles are more likely to request free services and less likely to use services with payment requirements. This indicates a need to subsidize access to care among the rural elderly, especially those in poor households. Poverty status is only related to use of services with payments, but household wealth is related to use of both free and paid services. This suggests that the household short-term economic status has less effect than long-term status on willingness to use care models. Whether the poorest quintile is less likely to use free services from a day care centre than the middle quintile, or is more likely than the poorer and middle quintiles to use services with full costs from a mobile team and day care centre is unknown.

"Three main limitations are lack of budget, knowledge and guidelines" was pointed out by a representative at the commune level. This implies that more must be learned about the implementation process. The current study identified a number of activities that would improve care models. However, information is limited about how to implement programs with constrained resources. Therefore, a well-designed pilot intervention is needed that focuses on the development of an intervention, the implementation process, and attitudes of users and providers.

Some methodological issues should be considered when interpreting and discussing this study. First, community-centric models of elderly care, especially mobile teams, are rare in Vietnam. Respondents had little or no experience in using or paying for such services. Therefore, pilot interventions are needed. Second, the analysis of willingness to use and pay for services is limited by certain socioeconomic determinants and does not currently cover the health issues that face the elderly. Third, ADLs do not cover all disability domains. Therefore, this assessment of elderly care needs may underestimate other care needs for functional impairments that were not assessed. Fourth, the economic data collected by the repeat census in 2007 may fluctuate by season in rural areas. Fifth, the study respondents were not provided with estimates of the prices. Most may have no experience in paying for these kinds of services. Thus, they might interpret levels of partial or full prices differently. Sixth, this cross-sectional survey could not detect causal relationships between willingness to use the service, ADL indices and socioeconomic factors.

## Conclusions

There is a demand for community-centric elderly care in rural Vietnam. Households expect to use services for their elderly to a greater extent than do the elderly themselves for different models and payment levels. Willingness to use services decreases when the potential fees are increased. The proportion of people who would require services is 2-3 times higher than the proportion of those willing to pay for services. Households are willing to pay more than their elderly members for day care and nursing centre services. The elderly were willing to pay more for mobile teams than were their households.

ADL index, age group, sex, educational level, marital status, living arrangement, household head status, living area, working status, poverty status, and household wealth are factors related to the willingness to use services. Despite many differences, there is overall agreement that community-centric elderly care will be used, and partly paid for, if it is provided by the government or associations. Network capacity building of health professionals and informal caregivers, as well as expanding support for the most vulnerable elderly through social security policies, is needed to assess which care services are essential for building and expanding care models. Pilot interventions are needed to scrutinize how these should be implemented in rural Vietnam.

## Abbreviations

ADL: Activities of daily living; NCD: Non-communicable diseases; USD: United States dollar; VND: Viet Nam dong; WTU: Willingness to use.

## Competing interests

The authors declare that they have no competing interests.

## Authors' contributions

LVH conceived and designed the research, developed its tools, supervised data collection, analysed data and drafted the manuscript. LL assisted during the conception phase, advised on the research design and data analyses, and revised the manuscript. NTKT, NVT and DVD advised on additional data analyses and revised the manuscript. NTKC advised on the research design, supervised data collection and revised the manuscript. KGS assisted in designing the qualitative research, analysing qualitative data, and drafting the manuscript. Each author read and approved the final manuscript.

## Pre-publication history

The pre-publication history for this paper can be accessed here:

http://www.biomedcentral.com/1472-6963/12/36/prepub
